# Is alcohol consumption the culprit for sarcopenia: Evidence from a cross-sectional and Mendelian randomization study

**DOI:** 10.1097/MD.0000000000044899

**Published:** 2025-10-03

**Authors:** Xiao-Feng Feng, Tao Sun

**Affiliations:** a The Second Clinical Medical College of Zhejiang Chinese Medical University, Hangzhou, Zhejiang, China; b Department of Hepatology, The Second Affiliated Hospital of Zhejiang Chinese Medical University, Hangzhou, Zhejiang, China.

**Keywords:** alcohol consumption, causality, Mendelian randomization analysis, National Health and Nutrition Examination Survey, sarcopenia

## Abstract

Alcohol is a relatively commonly consumed beverage worldwide. Inappropriate alcohol consumption can lead to problems such as alcoholic liver disease, upper gastrointestinal bleeding and osteoporosis. However, the effects of alcohol consumption on sarcopenia remain unknown. We aimed to provide evidence regarding the relationship between alcohol consumption and sarcopenia by analyzing data from the National Health and Nutrition Examination Survey (NHANES) in the United States and conducting Mendelian randomization (MR) analyses. Weighted multivariable-adjusted logistic regression was used to assess relationship using NHANES data from 1999 to 2006 and 2011 to 2016. Subsequently, a 2-sample MR study was conducted using pooled data from a genome-wide association study to determine the causal relationship. Sensitivity analysis was also used to confirm the robustness of the results. A total of 71,376 participants were enrolled in the NHANES observational study. Weighted multivariate-adjusted logistic regression analyses revealed that there was no significant correlation between alcohol consumption and sarcopenia (hardly drinking (odds ratio [OR] = 0.844; [95% confidence interval [CI], 0.842, 0.846]), slight drinking (OR = 0.745 [95% CI, 0.744–0.747]), binge drinking (OR = 0.896 [95% CI, 0.893–0.898]), heavy drinking (OR = 0.965 [95% CI, 0.962–0.967]), or alcoholism (OR = 0.974 [95% CI, 0.972–0.977])). The MR analysis revealed a causal relationship between alcohol consumption (OR = 2.112 [95% CI, 1.174–3.98]) and sarcopenia. The sensitivity analysis further confirmed the robustness and reliability of the results (all *P* > .05). Although cross-sectional studies could not determine whether alcohol consumption increases the risk of sarcopenia, a 2-sample MR analysis revealed that alcohol consumption is a risk factor for sarcopenia.

## 1. Introduction

Sarcopenia is a progressive and systemic skeletal muscle disease that is associated with aging.^[1]^ The clinical manifestations of this disease include reduced skeletal muscle mass, decreased muscle strength, and decreased physical function.^[2]^ These factors significantly increase the risk of adverse clinical outcomes among patients, including falls, fractures, disabilities, and death.^[3]^ Muscle atrophy can be divided into 2 categories: primary and secondary.^[4]^ Primary muscle atrophy occurs with age and is associated with mitochondrial dysfunction, neuromuscular damage, and decreased production or sensitivity of metabolic hormones. Secondary sarcopenia is typically attributed to systemic illnesses such as cancer, chronic kidney disease, diabetes,^[5]^ chronic obstructive pulmonary disease,^[6]^ and cirrhosis.^[7]^ It is speculated that approximately 50 million people worldwide are currently affected by sarcopenia, and the global prevalence of sarcopenia is expected to increase significantly in the coming decades.^[8]^ Sarcopenia can seriously damage the quality of life of patients and increase the population-level all-cause mortality rate. Furthermore, some studies have indicated that chronic alcohol consumption may contribute to sarcopenia.^[9]^ The current study presents evidence to support this association by analyzing data from the National Health and Nutrition Examination Survey (NHANES) and by conducting Mendelian randomization (MR) analyses.

It has been reported that 43% of the global population consumes alcohol, thus indicating that alcohol is a widely consumed beverage. However, alcohol abuse has become a significant risk factor for disease, disability, and death worldwide.^[10]^ For example, in the later stages of alcoholic liver disease, weight loss and significant loss of muscle mass can be observed among patients. Previous studies have indicated that 60% to 70% of patients with alcohol liver disease have some degree of sarcopenia.^[11,12]^ Sarcopenia has been detected in 20% to 60% of outpatients with alcohol-related cirrhosis, and the prevalence of sarcopenia in patients hospitalized for acute alcohol-related hepatitis is close to 100%.^[13]^ The presence of sarcopenia in alcohol-related cirrhosis patients also leads to worse outcomes, including decreased survival, decreased quality of life, variceal bleeding, ascites formation, hepatic encephalopathy, infections, longer hospital stays, and hepatorenal syndrome.^[13]^ The liver plays a central role in nutrient metabolism, including glucose homeostasis, protein synthesis, and drug/toxin metabolism. On the other hand, muscles have a strong influence on the liver and play a major role in all chronic liver diseases.^[14]^ Although there are many clinical problems associated with sarcopenia, there are still many gaps in knowledge on this disease.^[15]^ Furthermore, no preclinical human trials have clarified the effects of chronic excessive alcohol consumption on skeletal muscle protein metabolism and the subsequent onset of sarcopenia.^[16]^

The National Health and Nutrition Examination Survey an ongoing cross-sectional study conducted by the National Center for Health Statistics to assess the nutritional status of the U.S. population and emerging public health conditions.^[17]^ Therefore, the NHANES can provide high-quality, large-sample, and nationally representative data to assess the correlation between alcohol consumption and the risk of sarcopenia.^[18]^

MR analysis is an epidemiologic data analysis method that uses genetic variation as an instrumental variable for exposure to assess the causal relationship between exposure factors and outcome events.^[19]^ The methodology of MR studies is similar to that of randomized controlled trials because, during gamete formation, parental alleles are randomly assigned to offspring according to Mendelian laws, thus making MR studies equivalent to naturally occurring randomized controlled trials in a population.^[20]^ In addition, the results of MR studies are less susceptible to bias based on residuals or reverse causality because genetic variants are randomly assigned during meiosis and are thus independent of environmental factors.^[21]^

In this study, we comprehensively explored the relationship between alcohol consumption and sarcopenia by performing a large-scale observational study using NHANES data from 2 periods (1999–2006 and 2011–2016) and by conducting a 2-sample MR analysis.

## 2. Materials and methods

### 2.1. Study samples in the NHANES

The NHANES, a major program of the National Center for Health Statistics, is a nationwide, population-based nutrition and health survey conducted in the United States. The website provides information about the ongoing design of the NHANES survey, the informed consent forms signed by all study participants, and the fact that the National Center for Health Statistics Ethics Review Committee approved all study protocols prior to data collection. Since the data used in this study is anonymized and publicly available, approval from an institutional review board is not required.

**Figure 1.**
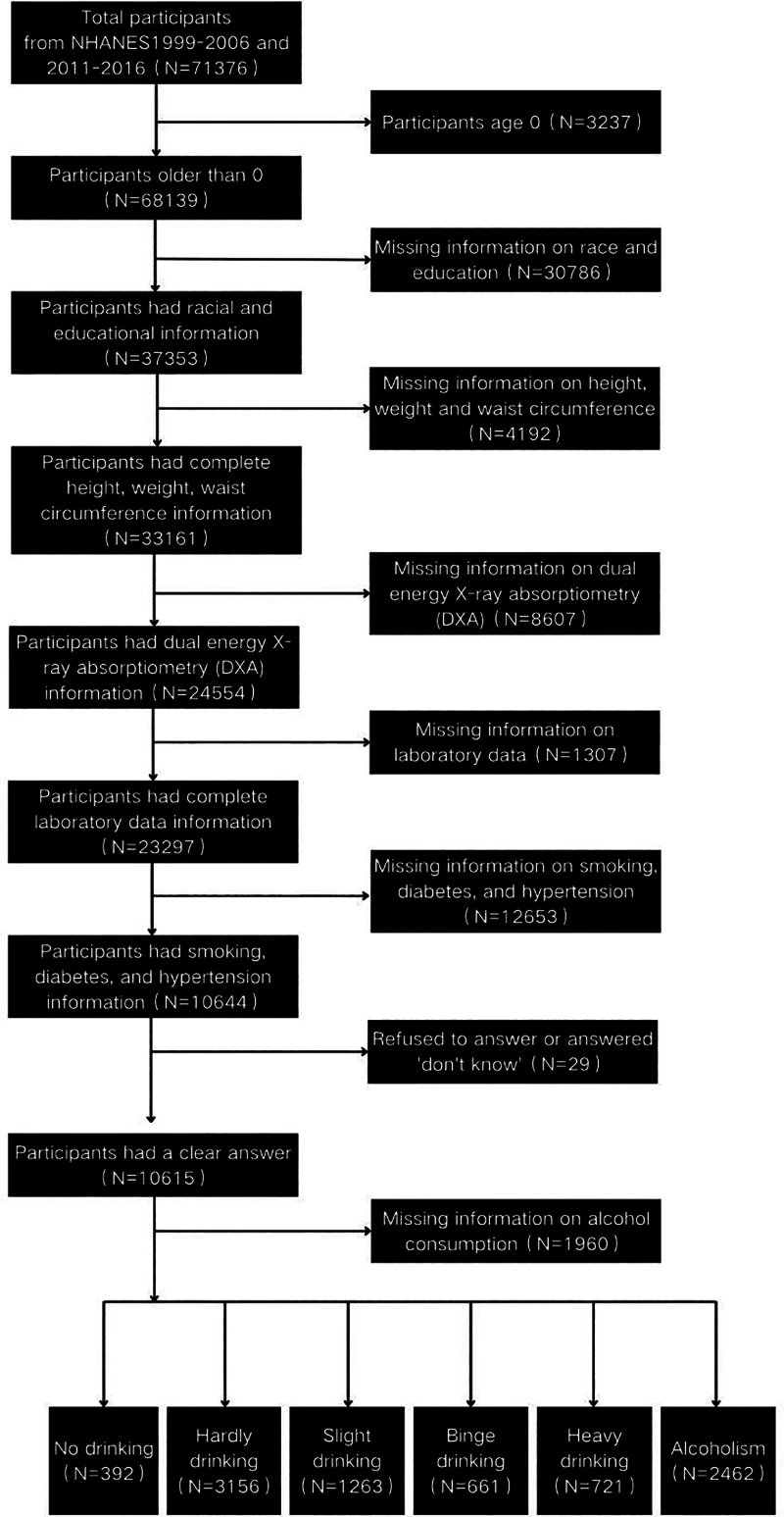
Study participant screening chart. DXA = dual-energy X-ray absorptiometry, NHANES = National Health and Nutrition Examination Survey.

Data from 2 time periods, i.e., 1999 to 2006 and 2011 to 2016, were examined in our study because the primary variable (dual-energy X-ray absorptiometry (DXA)) was assessed in these data cycles. A total of 71,376 participants were included in these time periods. We excluded the following participants: individuals aged 0 years (N = 3237); individuals lacking information on race and education (N = 30786); individuals lacking information on height, weight, and waist circumference (WC) (N = 4192); individuals lacking information on dual-energy X-ray absorptiometry (DXA), including those unable to participate in DXA examinations due to pregnancy within the past 7 days, obesity (>136 kg), height (>196 cm), or the use of radiographic contrast agents (e.g., barium).(N = 8607); (5) individuals lacking information on laboratory data (N = 1307); (6) individuals lacking information on smoking, diabetes, and hypertension questionnaires (N = 12653); (7) individuals who refused to answer or answered “don’t know” (N = 29); and (8) individuals lacking information on alcohol consumption (N = 1960). Therefore, ultimately, this study included 8655 participants, as shown in Figure 1.

### 2.2. Definition and assessment of alcohol use and sarcopenia in the NHANES

As reported in the NHANES data files, codebooks, and frequencies, 1 alcoholic beverage is defined as one 12-ounce glass of beer, one 4-ounce glass of wine or 1 ounce of liquor. By referring to other similar studies, the patterns of alcohol use are set here in 6 modes.^[22]^ “No drinking” is defined as not drinking any alcohol in one’s lifetime. “Hardly drinking” is defined as self-reported lifetime use or consumption of 12 or more alcoholic beverages in any form but no drinking habits or behaviors in the past 12 months. “Slight drinking” is defined as drinking at least 4/5 cups of alcohol once per year. “Binge drinking” is defined as drinking at least 4/5 cups of alcohol once a month. “Heavy drinking” is defined as drinking at least 4/5 cups of alcohol once a week. “Alcoholism” is defined as drinking at least 4/5 cups of alcoholic beverages every day or almost every day for one or more periods of a person’s life.^[23]^

Whole-body DXA was performed using a Hologic QDR-4500A fan-beam densitometer (Hologic, Inc., Bedford, Massachusetts). Participants who were pregnant within the past 7 days, obese (> 136 kg), tall (> 196 cm), or using radiographic contrast (barium) were unable to participate in the DXA. First, appendicular skeletal muscle mass (ASM) was defined as the sum of boneless lean muscle mass in the arms and legs. Subsequently, ASM was divided by body mass index (BMI) to further define sarcopenia.^[24]^ Because the target population for our study was U.S. adults from the NHANES database, we used the National Institutes of Health definition of sarcopenia (ASM/BMI < 0.789 for men and < 0.512 for women).

### 2.3. Other covariates used in the NHANES

To control for potential confounding effects, we referred to other studies and adjusted the analyses for demographic data, inspection data, laboratory data and questionnaire data.^[25]^ The demographic data included age (years, 20 to 85, median: 46), sex (male and female), race (Mexican American, Other Hispanic, Non-Hispanic White, Non-Hispanic Black, and Other Race – Including Multi-Racial), and education level (<9th grade, 9–11th grade (includes 12th grade with no diploma), high school graduation/general equivalency diploma or equivalent, some college or Associate of Arts degree, college graduate or above). The examination data included weight (kg), height (cm), WC (cm), and BMI (kg/m2). The laboratory data included blood urea nitrogen (mmol/L), total calcium (Ca, mmol/L), phosphorus (P, mmol/L), triglycerides (TG, mmol/L), total cholesterol (TC, mmol/L), creatinine (Cr. μmol/L) and uric acid (UA, μmol/L). The questionnaire data included information on smoking behavior (every day, some days, not at all), hypertension (yes/no), and diabetes (yes, no, borderline). The questions in the questionnaire were administered by trained interviewers using a computer-assisted personal interview system. The computer-assisted personal interview system has built-in consistency checks to reduce data entry errors. In addition, all data were reviewed by NHANES field office staff to ensure completeness, consistency, and analytical utility.

### 2.4. Genome-wide association study sources and single-nucleotide polymorphism selection

In this 2-sample MR analysis, the GWAS dataset was searched through the integrative epidemiology unit (IEU) OpenGWAS database (https://gwas.mrcieu.ac.uk/), with the keyword ‘ Alcohol drinker status: Current ‘ to obtain the ‘ ukb-d-20117 _ 2 ‘ dataset, which was used as the ‘ instrumental variable-exposure factor ‘ sample group (Sample: 360,726, single-nucleotide polymorphisms (SNPs): 13,586,591). Then, through the IEU OpenGWAS database, the GWAS dataset was searched with ‘ Whole body fat-free mass ‘ as the keyword to obtain the ‘ ukb-b-13354 ‘ dataset, which was used as the ‘ instrumental variable-outcome variable ‘ sample group (Sample: 454,850, SNPs: 9,851,867).

We preprocessed the data. The R package “TwoSampleMR” function extract_instruments was used to read the exposure factor (alcohol consumption) and screen the instrumental variables with the following criteria: *P* = 5*10^−8^ to find the instrumental variables that are significantly correlated with the exposure factor; clump = TRUE, instrumental variable for chained disequilibrium removed; *r*^2^ = 0.001; kb = 10000. After screening the instrumental variables, 3 instrumental variables (SNPs) related to the exposure factor (alcohol consumption) were obtained. We then read the outcome variable (sarcopenia) through the R package “TwoSampleMR” function extract_outcome_data and screened it against the instrumental variables in the exposure factors with the following metrics: proxies = TRUE; rsq = 0.8. None of the 3 instrumental variables (SNPs) associated with the exposure factor (alcohol consumption) were associated with the outcome variable (sarcopenia). Ultimately, data on exposure factors and outcome variables were combined to produce data that could be used in MR analyses.

### 2.5. Statistical analysis

In this observational study, we used a combination of cross-sectional and MR methods. To analyze the NHANES data, we used multivariate logistic-adjusted logistic regression to assess the relationships between different drinking patterns and sarcopenia. Five models adjusted for covariates were evaluated: model 1 was unadjusted; model 2 included sex, age, race, and education level; model 3 was adjusted for height, weight, BMI, and WC based on model 2; model 4 was adjusted for laboratory data based on model 3; and model 5 was adjusted for questionnaire data based on model 4. The results are expressed as odds ratios or βcoefficients (95% confidence intervals). Given the complex probabilistic clustering design of the NHANES, this study considered weights in multivariate logistic analysis.

For the 2-sample MR analysis, we used inverse variance weighting (IVW) as the primary method for assessing the causal relationship between alcohol consumption and sarcopenia risk. In addition, 4 complementary MR analysis methods, i.e., MR Egger, weighted median, simple mode, and weighted mode, were used to validate the IVW results. Because we used the SNP-exposure and SNP-outcome dual-sample approach, there may be different populations and different sequencing methods, which may lead to heterogeneity between samples and influence the analysis results. Therefore, to mitigate the effect of heterogeneity, we carried out a heterogeneity analysis. Next, to assess the presence of confounders in this study, we evaluated horizontal pleiotropy. In addition, to observe whether each instrumental variable (SNP) caused a significant change in the outcome situation, we performed sensitivity analysis via the leave-one-out method.

TwoSampleMR version 0.5.6 and IBM SPSS Statistics 25 (Chicago) were used to perform all the statistical analyses.

## 3. Results

### 3.1. Observational associations between alcohol consumption and the risk of sarcopenia in NHANES

#### 3.1.1. Screening of the study population

Table 1 shows the characteristics of the study participants. Based on the definition of sarcopenia, the study participants were divided into a sarcopenia group (961 patients) and a non-sarcopenia group (7694 patients). Patients with sarcopenia tend to be older, predominantly male, and non-Hispanic white. In addition, they had lower educational attainment and higher prevalence rates of hypertension and diabetes than the non-sarcopenia group. In addition, compared with the non-sarcopenia group, the sarcopenia group had higher body weight, WC, blood urea nitrogen levels, cholesterol levels, triglyceride levels, and uric acid levels as well as lower height, total calcium levels, total phosphorus levels, creatinine levels, and smoking rates.

**Table 1:** Demographic and clinical characteristics of participants with and without sarcopenia.

Items	Sarcopenia (n = 961)	Non-sarcopenia (n = 7694)	Statistics	*P*-value
Age (year)	60 (47.71)	44 (32.55)	−23.451	<.001*
Gender				
Male	676 (70.3%)	4677(60.8%)	33.060	<.001†
Female	285(29.7%)	3017(39.2%)		
Race				
Mexican American	365 (38.0%)	1161 (15.1%)	383.175	<.001†
Other Hispanic	68 (7.1%)	403 (5.2%)		
Non-Hispanic White	450 (46.8%)	4145 (53.9%)		
Non-Hispanic Black	46 (4.8%)	1495 (19.4%)		
Other race – including multi-racial	32 (3.3%)	490 (6.4%)		
Education level				
<9th Grade	264 (27.5%)	634 (8.2%)	355.888	<.001†
9–11th Grade (Includes 12th grade with no diploma)	162 (16.9%)	1378 (17.9%)		
High School Grad/GED or Equivalent	234 (24.3%)	2035 (26.4%)		
Some College or AA degree	204 (21.2%)	2349 (30.5%)		
College Graduate or above	97 (10.1%)	1298(16.9%)		
Weight (kg)	81.80 (70.65,95.85)	79.20 (67.80,92.20)	−5.259	<.001*
Height (cm)	163.40 (156.45,168.85)	171.00 (164.10,177.60)	−24.229	<.001*
WC (cm)	107.00 (97.85,118.20)	95.90 (85.90,106.00)	−21.632	<.001*
BMI (kg/m^2^)	30.99 (27.32,35.72)	26.93 (23.65,30.80)	−20.077	<.001*
Blood urea nitrogen (mmol/L)	5.00 (3.93,6.10)	4.28 (3.57,5.36)	−9.972	<.001*
Total calcium (mmol/L)	2.35 (2.30,2.40)	2.375 (2.300,2.425)	−3.538	<.001*
Cholesterol (mmol/L)	5.224 (4.551,5.922)	5.043 (4.396,5.775)	−4.898	<.001*
Phosphorus (mmol/L)	1.162 (1.033,1.292)	1.195 (1.070,1.324)	−4.807	<.001*
Triglycerides (mmol/L)	1.626 (1.185,2.399)	1.332 (0.892,2.066)	−9.992	<.001*
Uric acid (µmol/L)	345.00 (291.50,404.50)	321.20 (267.70,380.70)	−8.092	<.001*
Creatinine (µmol/L)	70.72 (61.88,88.40)	76.91 (61.90,88.40)	−2.390	.017*
Smoking				
Every day	273 (28.4%)	3408 (44.3%)	110.374	<.001†
Some days	83 (8.6%)	806 (10.5%)		
Not at all	605 (63.0%)	3480 (45.2%)		
Hypertension				
Yes	423 (44.0%)	2199 (28.6%)	96.392	<0.001†
No	538 (56.0%)	5495 (71.4%)		
Diabetes				
Yes	158 (16.4%)	558 (7.3%)	124.921	<.001†
No	767 (79.8%)	7022 (91.3%)		
Borderline	36 (3.7%)	114 (1.5%)		
Type of alcohol consumption				
No drinking	71 (7.4%)	321 (4.2%)	60.847	<.001†
Hardly drinking	355 (36.9%)	2801 (36.4%)		
Slight drinking	98 (10.2%)	1165 (15.1%)		
Binge drinking	46 (4.8%)	615 (8.0%)		
Heavy drinking	64 (6.7%)	657 (8.5%)		
Alcoholism	327 (34.0%)	2135 (27.7%)		
Age (year)	60 (47.71)	44 (32.55)	−23.451	<.001*

AA = associate of arts, BMI = body mass index, GED = general equivalency diploma, WC = waist circumference.

Data were presented as median (interquartile range) or n (%).

* Mann–Whitney *U* test for continuous variables.

† Pearson chi-squared test for categorical variables.

#### 3.1.2. Association between alcohol consumption and sarcopenia based on the NHANES data

Weighted multivariate logistic regression analysis revealed that there was no significant association between alcohol consumption and the risk of sarcopenia (Table 2). In the unadjusted model, there was a negative association between alcohol consumption and sarcopenia. After adjusting for age, sex, race, and education, a negative association was still observed between alcohol consumption and sarcopenia. However, after further adjusting for height, weight, BMI, and WC, the correlation between alcohol consumption and sarcopenia became less significant. Moreover, after even more adjustment, no significant relationship was found between alcohol consumption and sarcopenia.

**Table 2:** Weighted multivariate logistic regression analysis: association between alcohol consumption categories and sarcopenia.

Items	Model 1: OR (95% CI)	Model 2: OR (95% CI)	Model 3: OR (95% CI)	Model 4: OR (95% CI)	Model 5: OR (95% CI)
No drinking	Reference	Reference	Reference	Reference	Reference
Hardly drinking	0.494 (0.493–0.495)	0.580 (0.579–0.581)	0.855 (0.853–0.857)	0.858 (0.856–0.860)	0.844 (0.842–0.846)
Slight drinking	0.286 (0.286–0.287)	0.472 (0.471–0.472)	0.780 (0.778–0.782)	0.770 (0.768–0.772)	0.745 (0.744–0.747)
Binge drinking	0.267 (0.266–0.267)	0.424 (0.423–0.425)	0.920 (0.917–0.922)	0.911 (0.909–0.914)	0.896 (0.893–0.898)
Heavy drinking	0.387 (0.386–0.388)	0.601 (0.600–0.603)	0.980 (0.977–0.982)	0.980 (0.977–0.983)	0.965 (0.962–0.967)
Alcoholism	0.566 (0.565–0.567)	0.637 (0.636–0.638)	0.961 (0.959–0.964)	0.976 (0.974–0.978)	0.974 (0.972–0.977)

BMI = body mass index, CI = confidence interval, OR = odds ratio.

Model 1 adjusted for: none. Model 2 adjusted for: gender, age, race, and education level. Model 3 was adjusted for height, weight, BMI, and waist circumference based on the factors included in Model 2. Model 4 was adjusted for factors included in Model 3 and adjusted for blood urea nitrogen, total calcium, total cholesterol, creatinine, phosphorus, triglycerides, and uric acid. Model 5 was adjusted for factors included in Model 4 and adjusted for hypertension status, diabetes status, and smoking status.

### 3.2. Causal relationship between alcohol consumption and the risk of sarcopenia measured by MR analysis

Although no significant correlation was observed between alcohol consumption and sarcopenia in the multivariate logistic regression analyses described above, evidence of a correlation between the 2 can be found in clinical practice and literature.. Therefore, we hypothesized that alcohol consumption is a risk factor for sarcopeni and conducted MR analysis to determine whether there is a causal relationship between alcohol consumption and the risk of sarcopenia. Because all MR studies have an inherent challenge of possible directed pleiotropy, this cannot be completely eliminated.^[26]^ Therefore, during our MR analyses, in addition to the primary IVW method, other complementary MR methods also provided equally consistent results. Moreover, we also performed sensitivity analyses to better rule out the presence of pleiotropy and provide more robust support for the causal effect estimates of our results. The R package “TwoSampleMR” function harmonise_data unifies the effect alleles and effect sizes, and the MR function combines 5 algorithms (MR Egger, weighted median, inverse variance weighted, simple mode, weighted mode) to perform the MR analysis. As shown in Table 3, the results indicate that the odds ratio (effect value) using the IVW method is >1, indicating that alcohol consumption significantly affects sarcopenia and that alcohol consumption is a risk factor for sarcopenia. Furthermore, these results are consistent with those of other complementary MR analyses in terms of the direction of the causal estimates and the magnitude of the causal effects, thus suggesting that these results are reliable and robust.

**Table 3:** MR estimates for each method for assessing the causal effect of alcohol consumption on the risk of sarcopenia.

Outcome	Exposure	Method	Number of SNPs	b	se	*P*val	OR (95% CI)
Whole-body fat-free mass	Alcohol drinker status: Current	MR Egger	3	1.572	0.500	.196	4.817 (1.808–12.835)
		Weighted median	3	0.798	0.299	.008	2.222 (1.215–4.064)
		Inverse variance weighted	3	0.747	0.300	.013	2.112 (1.174–3.798)
		Simple mode	3	0.913	0.300	.169	2.491 (1.062–5.843)
		Weighted mode	3	0.932	0.456	.178	2.539 (1.102–5.850)

CI = confidence interval, MR = Mendelian randomization, OR = odds ratio, SE = standard error, SNPs = single-nucleotide polymorphisms.

**Figure 2.**
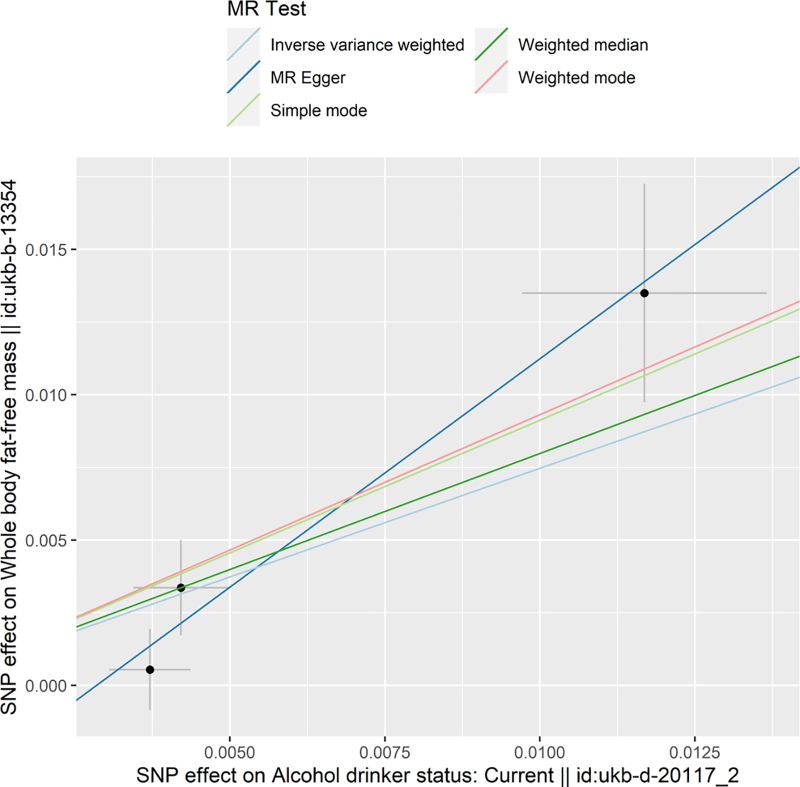
Scatterplot. MR = Mendelian randomization, SNPs = single-nucleotide polymorphisms.

To determine the correlation between exposure factors and outcomes, we combined the SNP-exposure factor effect and the SNP-outcome effect to construct correlation scatter plots, as shown in Figure 2. The IVW algorithm revealed that the effect of the instrumental variable (SNP) on the exposure factor (alcohol consumption) and the effect of the instrumental variable (SNP) on the outcome (sarcopenia) were positively correlated, thus providing further evidence that alcohol consumption is a risk factor for sarcopenia.

**Figure 3.**
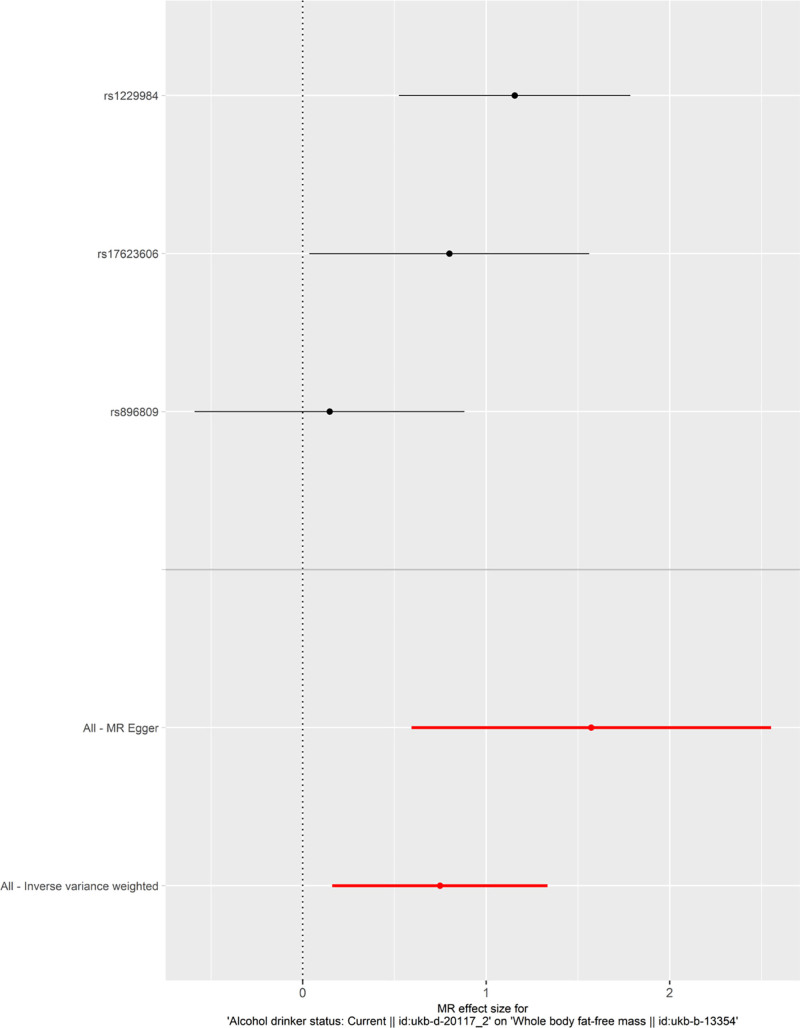
Forest plot. MR = Mendelian randomization.

To determine the diagnostic efficacy of the predicted exposure factors for each SNP locus on the outcome diagnosis, we constructed a forest plot combining the risk effects of the estimated exposure factors for each SNP on the outcome, as shown in Figure 3. The IVW method revealed that the overall effect value of exposure factors on outcome variables was >0, thus indicating that alcohol consumption increases the risk of sarcopenia.

**Figure 4.**
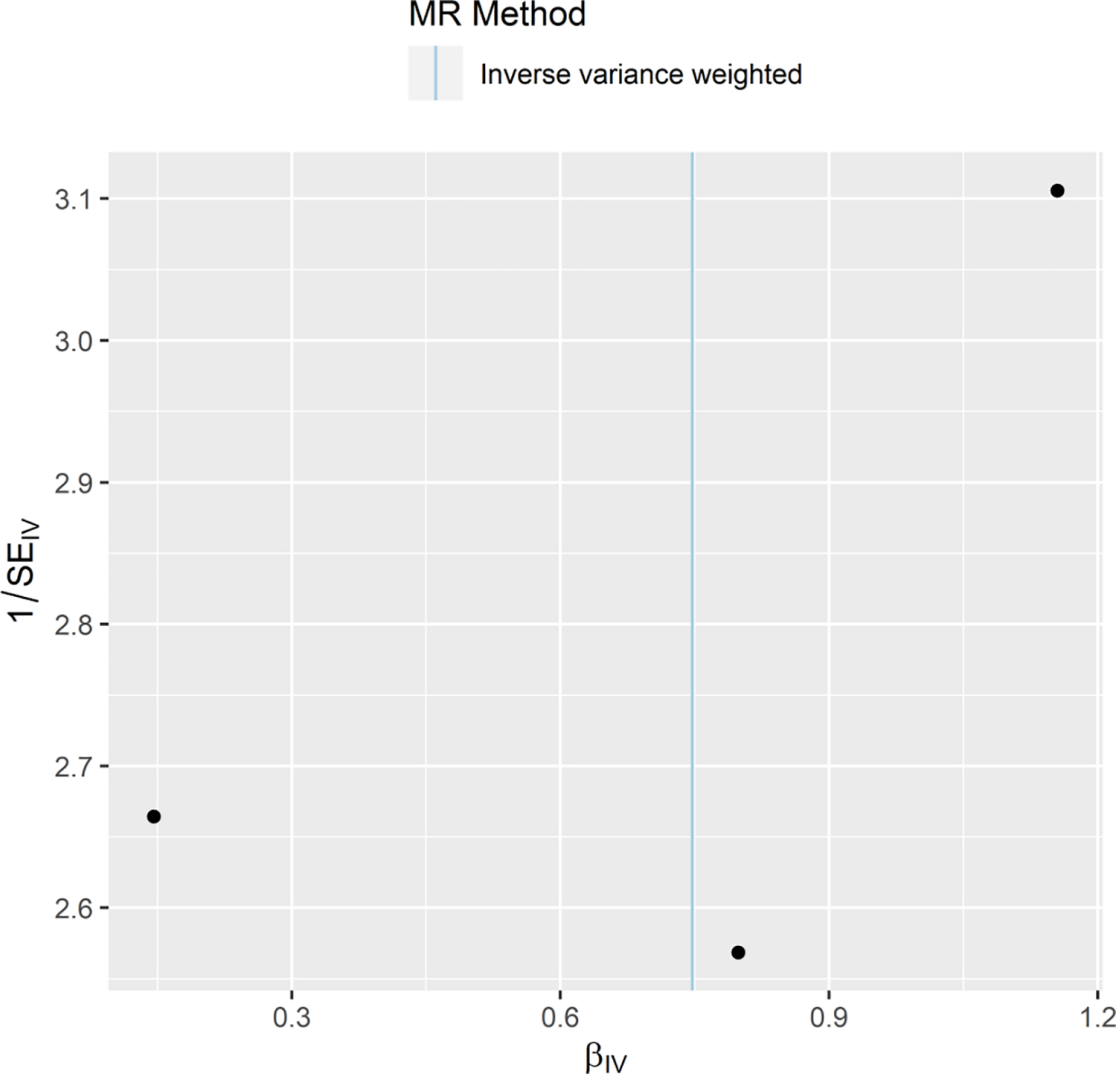
Funnel plot. MR = Mendelian randomization, SE = standard error.

To determine whether this analysis is stochastic and whether MR conforms to Mendel second law of random grouping, we combined the β and standard error of each instrumental variable in a funnel plot, as shown in Figure 4. The instrumental variables of MR as a whole are symmetrically distributed on the left and right sides of the IVW line, suggesting that the forward MR analysis conforms to stochasticity.

To assess the reliability of the MR analysis results, we performed sensitivity analyses of the MR results, which mainly included heterogeneity tests, horizontal pleiotropy tests, and the leave-one-out method.

To check the effect of heterogeneity, we analyzed heterogeneity using the mr_heterogeneity function of the R package “TwoSampleMR.” The results are shown in Table 4, in which the Q_*p*val values are >.05, indicating that there is no heterogeneity between the 2 groups of samples.

**Table 4:** Results of the MR heterogeneity test.

Outcome	Exposure	Method	Q	Q_df	Q_pval
Whole-body fat-free mass	Alcohol drinker status: Current	MR Egger	0.906	1	.341
		Inverse variance weighted	4.189	2	.123

MR = Mendelian randomization.

We conducted a horizontal pleiotropy test using the R package “TwoSampleMR” function mr_pleiotropy_test. The results are shown in Table 5. The *P*-value was >.05, indicating that there was no horizontal pleiotropy, i.e., there are no confounding factors in the study, which further highlights the reliability of the results.

**Table 5:** Horizontal pleiotropy test.

Outcome	Exposure	Egger_intercept	se	*P*val
Whole-body fat-free mass	Alcohol drinker status: current	−0.004	0.002	.321

**Figure 5.**
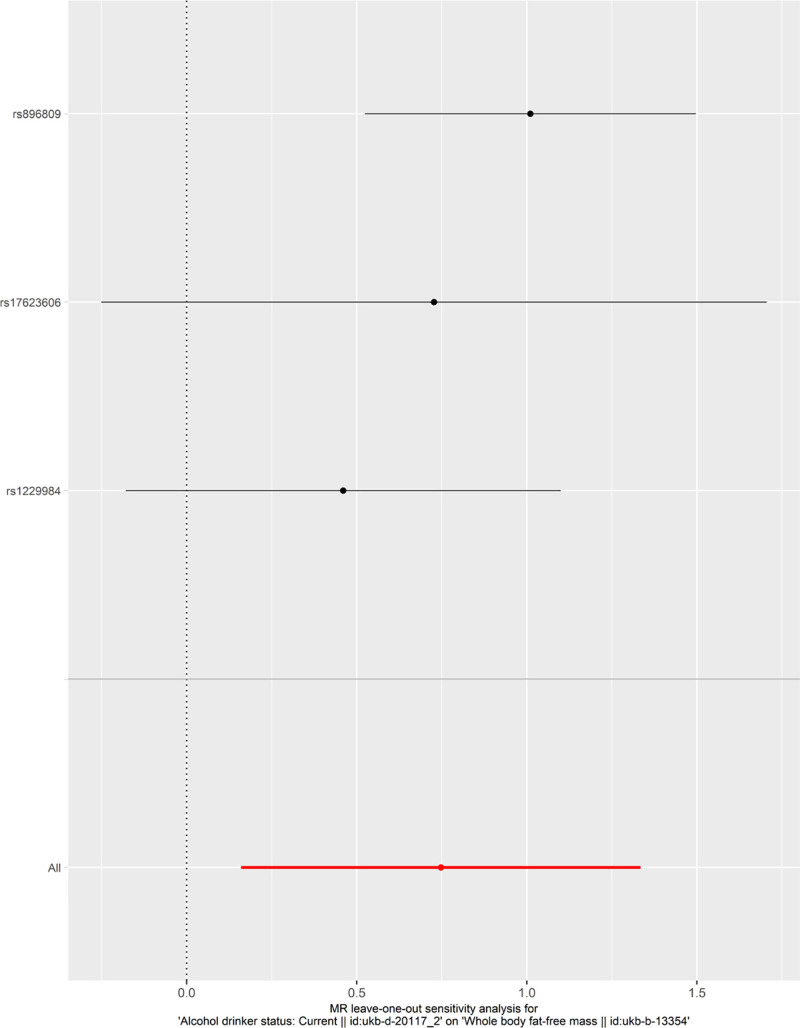
Sensitivity analysis via the leave-one-out method. MR = Mendelian randomization.

We performed sensitivity analysis via the leave-one-out method using the R package TwoSampleMR function mr_leaveoneout, as shown in Figure 5. The aim of this analysis was to exclude each SNP one at a time and then calculate the meta effect of the remaining SNPs to determine whether the results changed after the exclusion of each SNP. The changes in the results were analyzed using an inverse-direction weighted penalty, and a forest plot was constructed to visualize the results. The sensitivity analysis revealed that our MR analysis results were robust and reliable.

## 4. Discussion

In this study, we performed an observational study based on data from the nationally representative NHANES 1999 to 2006 and 2011 to 2016 cohorts as well as a 2-sample MR analysis to investigate the relationship between alcohol consumption and sarcopenia. Our findings suggested that there was no significant relationship between alcohol consumption and sarcopenia in the NHANES observational study, but a causal effect of alcohol consumption on sarcopenia was confirmed via the 2-sample MR approach, suggesting that alcohol consumption is a risk factor for sarcopenia.

Numerous studies have shown that alcohol consumption has a major influence on the development of sarcopenia. Studies have shown that chronic excessive alcohol consumption may lead to dysbiosis and autophagy-induced hyperammonemia in the intestinal flora, leading to sarcopenia through the activation of muscle growth inhibitors, adenosine 5‘-monophosphate (AMP)-activated protein kinase and Regulated in Development and DNA damage response 1, initiating the upregulation of muscle proteolysis, the downregulation of muscle protein synthesis, and the inactivation of Insulin-like growth factor 1.^[2,16]^ Similarly, cross-sectional studies have shown that higher alcohol intake (both in terms of quantity and frequency) is associated with an increased risk of low muscle mass and low muscle strength.^[27]^ A prospective population-based study revealed a significant positive association between alcohol consumption and muscle strength loss in Japanese individuals, and this relationship did not change over 2 years.^[28]^ However, another study reported inconsistent results regarding the association of alcohol consumption with both muscle mass and muscle strength.^[29]^ In addition, in a systematic evaluation and meta-analysis of population-based studies examining the relationship between sarcopenia and alcohol consumption, we found that in a meta-analysis of 19 observational studies, there was no significant relationship between alcohol consumption and the risk of sarcopenia.^[30]^ An early meta-analysis revealed no evidence that alcohol consumption increases the risk of sarcopenia.^[31]^ Herein, a cross-sectional study using data from the NHANES revealed no significant correlation between alcohol consumption and sarcopenia. Our findings are consistent with the results mentioned above, which showed no significant association between the 2.

However, in the later stages of alcoholic liver disease, we can clearly see the development of sarcopenia in the clinic. The association between alcohol consumption and sarcopenia is complex; therefore, purely cross-sectional analyses of populations may not yield convincing conclusions.^[32]^ Therefore, in addition to using nationally representative observational studies, we have taken the MR approach further to elucidate the causal relationship between alcohol consumption and sarcopenia. According to the MR analysis, alcohol consumption is a very important risk factor for the development of sarcopenia. In addition to the main IVW method, the supplementary MR method is consistent in terms of the direction of causal estimation and the magnitude of causal effects. At the same time, sensitivity analysis further confirms that our MR analysis results are robust and reliable.

Regarding the inconsistencies between the cross-sectional findings and the conclusions of the MR analysis, we believe that several factors may be at play. For example, we used self-report questionnaire data from the NHANES, which may be subject to recall bias and social desirability bias^[33]^; among the 2 cohorts we chose, although the overall sample size was larger, the sample size for sarcopenia was smaller, so the experimental results may have been influenced by chance; not all factors contributing to sarcopenia were listed in intake, physical activity, pharmacological interventions, chronic liver disease, etc, which may have biased the results^[34]^; for the definition of drinking, we only considered the total amount of alcohol consumed and did not consider the specific amount consumed each time, so we may have overlooked the importance of the amount consumed per drinking occasion in drinking patterns. In summary, the NHANES study design is cross-sectional, which means it cannot establish a causal relationship between alcohol consumption and sarcopenia, and such a study design has a high potential for residual confounding. In contrast, the MR method is more likely to reduce concerns about confounding variables and reverse causality, as genetic variants are fixed at conception and have less association with confounding factors compared to directly measured environmental exposures.

The major strength of this study is the combination of the observational NHANES study with MR methods. The combined assessment of factors and large-sample sizes allowed us to reliably adjust for the effects of multiple confounders simultaneously in multivariate regression models and provided sufficient statistical power to assess the relationship between alcohol consumption and sarcopenia. In addition, the MR approach avoids unmeasured confounders as well as reverse causality bias. It is important to note that the 2 methods used in the present study yielded inconsistent results, thus causing us to think about the possible association between alcohol consumption and sarcopenia. However, there are several limitations to our study. First, the source of data for our cross-sectional study was limited to the United States; therefore, the generalizability of our findings to other racial groups may be limited. Second, cross-sectional studies provide a lower level of evidence than cohort studies and thus lack particularly good persuasive evidence for the results of cross-sectional studies. Third, owing to the limited number of SNPs for alcohol consumption – which may have some impact on the statistical efficacy of MR analyses, even with a large-sample size and strong instrumental variables – our findings should still be interpreted with caution. Further research is needed to elucidate the association between alcohol consumption and sarcopenia.

In conclusion, our study explored the relationship between alcohol consumption and sarcopenia, but some aspects warrant further exploration. To address the limitations of the current study and provide stronger evidence, large-scale, multiethnic, and prospective studies need to be conducted. In addition, as our understanding of the effects of alcohol consumption on the underlying mechanisms of sarcopenia grows, we need to consider the effects of other factors, such as the type of alcohol, how it is produced, and the rate at which it is consumed, in future studies.

## Acknowledgments

We sincerely thank the IEU OpenGWAS database for providing the GWAS data and the researchers and participants of the NHANES for the data collection and management of the data resources. We thank Zhongke Shengxin for reviewing and verifying the data.

## Author contributions

**Conceptualization:** Tao Sun.

**Data curation:** Xiao-Feng Feng.

**Funding acquisition:** Tao Sun.

**Investigation:** Xiao-Feng Feng.

**Methodology:** Xiao-Feng Feng.

**Software:** Xiao-Feng Feng.

**Supervision:** Xiao-Feng Feng.

**Validation:** Tao Sun.

**Visualization:** Xiao-Feng Feng.

**Writing – original draft:** Xiao-Feng Feng.

**Writing – review & editing:** Tao Sun.
